# Evaluation of Pentraxin 3 and Serum Amyloid A in the Gingival Crevicular Fluid of Patients with Periodontal Disease and Obesity

**DOI:** 10.3390/jcm12103523

**Published:** 2023-05-17

**Authors:** Dora Maria Popescu, Dorin Nicolae Gheorghe, Adina Turcu-Stiolica, Andrada Soancă, Alexandra Roman, Claudiu Marinel Ionele, Eduard Mihai Ciucă, Virgil Mihail Boldeanu, Lidia Boldeanu, Allma Pitru, Petra Șurlin

**Affiliations:** 1 Department of Periodontology, Faculty of Dental Medicine, University of Medicine and Pharmacy of Craiova, 200349 Craiova, Romania; popescudoramaria@yahoo.com (D.M.P.); dorinngheorghe@gmail.com (D.N.G.); 2 Department of Pharmacoeconomics and Statistical Analysis, Faculty of Pharmacy, University of Medicine and Pharmacy of Craiova, 200349 Craiova, Romania; adina.turcu@gmail.com; 3 Department of Periodontology, Faculty of Dental Medicine, Iuliu Hațieganu University of Medicine and Pharmacy Cluj-Napoca, Victor Babeș St., No. 15, 400012 Cluj-Napoca, Romania; 4 Department of Gastroenterology and Hepatology, Faculty of Medicine, University of Medicine and Pharmacy of Craiova, 200349 Craiova, Romania; ioneleclaudiu@gmail.com; 5 Department of Oral and Maxillofacial Surgery, Faculty of Dental Medicine, University of Medicine and Pharmacy of Craiova, 200349 Craiova, Romania; ciucaeduard@yahoo.com; 6 Department of Immunology, Faculty of Medicine, University of Medicine and Pharmacy of Craiova, 200349 Craiova, Romania; mihailvirgilboldeanu@gmail.com; 7 Department of Microbiology, Faculty of Medicine, University of Medicine and Pharmacy of Craiova, 200349 Craiova, Romania; barulidia@yahoo.com; 8 Department of Oral Pathology, Faculty of Dental Medicine, University of Medicine and Pharmacy of Craiova, 200349 Craiova, Romania; allmapitru75@yahoo.com

**Keywords:** periodontal disease, obesity, gingival crevicular fluid, pentraxin 3, serum amyloid A

## Abstract

Background: Pentraxin 3 (PTX3) is associated with periodontal tissue inflammation, a condition that precedes alveolar bone resorption. It is also elevated in obese tissues and is a useful biomarker of proinflammatory status. Serum amyloid A (SAA) is a proinflammatory and lipolytic adipokine. Adipocytes strongly express SAA, which suggests that it may have a significant role in the production of free fatty acids and local and systemic inflammation. Materials and Methods: We statistically analyzed the gingival crevicular fluid (GCF) values of PTX3 and SAA in patients with periodontal disease, who were diagnosed with obesity, and compared them with the values of inflammatory markers from patients diagnosed with one of the diseases and with healthy patients. Results: The patients with obesity and periodontitis had significantly higher levels of PTX3 and SAA than the patients diagnosed with either obesity or periodontitis. Conclusions: These two markers are involved in the association between the two pathologies, as evidenced by the correlations between these levels and some clinical parameters.

## 1. Introduction

Periodontal disease (PD), an inflammatory condition, involves microbial dental plaque, genetic factors, and environmental factors. Increased levels of lymphocytes, neutrophils, and macrophages, which are detectable in early lesions, are specific markers of PD progression [1]. A variety of systemic risk factors, in addition to the etiology, contributes to the development of PD by modulating the patient’s immune response and influencing the progression of inflammation [2]. A major metabolic and nutritional disease, obesity has been considered a risk factor for several diseases with a systemic impact on the human body, including cardiovascular disease, hypertension, osteoarthritis, and diabetes. It is also associated with increased susceptibility to bacterial infections [3].

The prevalence of adult obesity has tripled globally over the past three decades and causes a higher body fat percentage, which may lead to chronic low-grade inflammation with few or no symptoms [4]. Body mass index (BMI) is the most accepted method for evaluating obesity [5]. Underweight (less than 18.5 kg/m^2^), normal (18.5 to 24.9 kg/m^2^), overweight (25.0 to 29.9 kg/m^2^), and obese (over 30.0 kg/m^2^) are the four categories for BMI, that are determined by dividing the weight in kilograms by the square height in meters. The waist circumference, waist-to-hip ratio (the dimensionless ratio of the circumference of the waist divided by the hips), and overall body fat are additional measures of obesity [6].

The comorbidity between obesity and periodontitis was noticed by the new 2017 World Workshop Classification (WWC 2017) of periodontal diseases and conditions, which identified obesity as a serious metabolic issue due to various factors related to the loss of periodontal tissues and an increased risk of periodontitis in obese people [7]. BMI strongly correlates with periodontal attachment loss severity in those with periodontal disease, and obesity is linked to deep periodontal pockets.

Inflammatory mediators, cytokines, leukocytes, enzymes, organic ions, tissue breakdown products, proteins, and a variety of other components are found in gingival crevicular fluid (GCF). Adipokines and cytokines are significantly altered in dysfunctional and excessive adipose tissue during the development of obesity. Increased levels of proinflammatory cytokines in GCF and serum in obese subjects suggest local inflammation [8].

Periodontal disease can be correctly defined and managed by detecting oral dysbiosis and changes in the metabolic product. The crevicular fluid released at the gingival sulcus is a serum transudate or inflammatory exudate and is considered to have a variety of roles such as antibacterial, antifungal, and mechanical cleansing of the sulcus [9]. During any periodontal infection, the levels of the host defense and the proinflammatory mediator components detected in saliva and gingival sulcus fluid increase. Neutrophils, antibodies, complement proteins, cytokines, and other microbial and host products can all be identified in the GCF [10].

Pentraxins (PTX), which correspond to a family of evolutionary components that also includes lipopolysaccharides and membrane proteins and is related to cytokines such as TNF-and IL-1, are classical mediators of inflammation and markers of acute phase reactions [11]. Pentraxin 3 (PTX3), also known as Tumor necrosis factor-inducible gene 14 protein (TSG-14), is the first type of long Pentraxin identified as belonging to the PTX family and a significant independent indicator of disease activity. To the periodontal level, PTX3 analysis in GCF or plasma can help to identify the risk of destructive or degenerative diseases. In patients with severe chronic disease, the level of PTX3 in the blood may thus be highly associated with the severity of infection [12].

The PTX family member serum amyloid A (SAA), has a role in immunity, inflammation, and perhaps periodontal inflammation. For many decades, elevated levels of circulating SAA have been identified as a risk factor for certain chronic inflammatory diseases [13]. Obesity, metabolic syndrome, and diabetes have all been associated with long-term and modest increases in SAA concentrations. It is possible that this biomarker could be used as a therapeutic target for treating obesity-related amyloidosis and the amyloid deposits that result from systemic amyloidosis. It has been demonstrated that high levels of SAA in the blood correlate with body fat [14], and weight loss can reduce these levels [15]. As an apolipoprotein and an inflammatory protein, SAA has been the subject of cross-sectional and prospective research; weight loss interventions have examined the connection in recent years between SAA and obesity and have developed interesting results [16].

Aim: This research aims to determine the GCF values of PTX3 and SAA in patients with periodontal disease diagnosed with obesity and test for the possible correlations between these values and certain clinical parameters.

## 2. Materials and Methods

### 2.1. Study Design

The present study was conducted after the approval of the Ethics Commission of the University of Medicine and Pharmacy of Craiova, respecting the requirements regarding the completion of consent forms for each patient. We also complied with the European Union’s General Data Protection Regulation (GDPR) and the Declaration of Helsinki 1975–2013 for data protection and patient privacy. The research was conducted from October 2022 to February 2023.

### 2.2. Patient Selection

The patients were selected from the Diabetes and Nutritional Diseases, Gastroenterology and Hepatology Clinics, and the Periodontal Department of the University of Medicine and Pharmacy of Craiova, where they were medically and periodontally examined. The inclusion criteria were patients aged ≥ 18, regardless of sex. The exclusion criteria were (i) anti-inflammatory or antibiotic medication in the last 30 days prior to the initial sampling of the gingival crevicular fluid; (ii) other systemic diseases, (iii) smoking, and (iv) pregnancy.

The periodontal evaluation was performed in the Periodontology Clinic of the University of Medicine and Pharmacy of Craiova. The periodontitis diagnosis [17] was established according to WWC 2017 new classification.

The sample of our study was represented by 55 patients, assigned to one of four groups, as follows: (i) patients with obesity: 14 patients (O group); (ii) patients with periodontitis: 14 patients (P group); (iii) patients with obesity and periodontitis: 15 patients (P + O group); (iv) healthy patients, control group: 12 patients (C group).

In the statistical analysis, we used the PPD and GI values from the periodontal chart. Moreover, the BMI values were retained from the medical charts of patients for statistical analysis.

The periodontal evaluation was performed with a UNC15 periodontal probe (Medesy, Maniago, Italy) for each patient, by the same well-trained dentist (DMP). Except for the third molars and any remaining root tips, all teeth were examined in six sites (mesio-vestibular, centro-vestibular, disto-vestibular, mesio-lingual, centro-lingual, and disto-lingual), recording the immediate full millimeter. For the recorded variables, PPD and GI, the measurements were assessed as follows: the PPD was measured in millimeters for each patient and was obtained by summing the measured values and dividing them by the number of examined sites; the GI was established for each patient as the average for each group. According to Löe [18], the GI is assessed from 0 to 3 with 0 to 1.0 for gingival inflammation considered mild, 1.1 to 2.0 for gingival inflammation considered moderate, and >2.0 for gingival inflammation considered severe.

### 2.3. Gingival Crevicular Fluid Sampling

Two GCF samples from each participant, with a one minute interval between the collection of each sample, were collected from the tooth with the deepest periodontal pocket or from the gingival sulcus for periodontally healthy patients, following the clinical periodontal evaluation. Each tooth was isolated using cotton rolls and then dried, as a precaution against sample contamination. The supragingival plaque was eliminated. The paper strips (PerioPaper, Oraflow Inc., Smithtown, NY, USA) were inserted within the periodontal pocket or sulcus until mild resistance was felt and kept in place for 30 s. Upon removal, the strips were visually inspected for blood stains and dipped into a plastic microtube containing saline buffer solution (PBS). The samples were conserved at −20 °C until they were used for immunological assessment.

### 2.4. Immunological Assessment

For immunological assessment, we used the commercially available tests for enzyme-linked immunosorbent assay (ELISA), Human SAA ELISA Kit, range1.25–80 ng/mL, and Human PTX 3/TSG-14 ELISA Kit, range 6.86–5000 pg/mL, in accordance with the manufacturer’s instructions (Elabscience, Biotech, Houston, TX, USA). During the procedure, a common optical analyzer with a 450 nm wavelength was employed.

### 2.5. Statistical Analysis

The results were expressed as the mean, standard deviation (SD), and median (interquartile range, IQR) to describe continuous variables. Using GraphPad 9.5.1. Software (LLC, San Diego, CA, USA), we checked whether the data were normally distributed with the Kolmogorov–Smirnov test and performed a one-way ANOVA (if normally distributed) or Kruskal–Wallis test (if not normally distributed) to test the differences among the groups. If differences were observed, we performed the post-test (Dunn’s multiple comparison test). The existence of significant correlations between the different datasets in the P + O group was assessed using Spearman’s coefficients (−1 < rho < 1) and visually presented with the correlation heatmap matrix (color range from bright blue for strong positive correlations to bright red for strong negative correlations). The power analysis of our results was performed using G*Power 3.1.9.7, at a power factor of 80% for each of the four groups, assuming an alpha level of 0.05. The results yielded an achieved power between 78% and 99% for the different analyses. A *p*-value < 0.05 was set as statistically significant.

## 3. Results

The patient’s mean age was 38.33 ± 3.52, 51.9% were female, 48.1% were male, and the living environment was predominantly urban, 80% (44 patients from an urban environment, 11 patients from a rural environment).

### 3.1. Comparisons between Groups

#### 3.1.1. Comparisons of Demographic and Clinical Parameters between Groups

No differences in age (*p*-value = 0.2578) or sex (*p*-value = 0.9914) were observed among the four groups.

The mean BMI was 33.04 ± 1.10 for the O group and 32.75 ± 1.14 for the P + O group, but the difference was not statistically significant (*p* = 0.68).

The PPD values were higher in the P + O group compared to the P group (1.28 fold), with a statistically significant difference (*p* = 0.0156).

The GI values were higher in the P + O group (1.15 fold than group P, and 2.25 fold than group O), but the statistically significant difference was with the O group (*p* = 0.0025). (Table 1).

#### 3.1.2. Comparisons of the Immunological Parameters between Groups

The SAA value (Table 2) was higher in all the test groups than in the C group with a statistically significant difference (*p* < 0.0001).The highest values were in the P + O group (3.16 fold than group P, 4.75 fold than group O, and 8.41 fold than group C), with significant differences (*p* < 0.0001).

The PTX3 values (Table 3) were higher in all the test groups than in group C with a statistically significant difference (*p* < 0.0001).The highest values were in the P + O group (1.32 fold than group P, 1.96 fold than in group O, and 2.66 fold than group C), with significant differences (*p* < 0.0001).

### 3.2. Correlations between the Parameters for the P + O Group

The SAA values were weakly positively correlated to the limit of significance with the BMI (rho = 0.341, *p* = 0.05). A statistically significant strong correlation between the SAA and PPD (rho = 0.615, *p* < 0.0001) and a moderate statistically significant positive correlation between the SAA and GI (rho = 0.490, *p* < 0.0001) were found.

The PTX3 level correlated strongly and significant with the GI (rho = 0.732, *p* < 0.0001) and very strongly and significant with the PPD (rho = 0.861, *p* < 0.0001).

**Figure 1:**
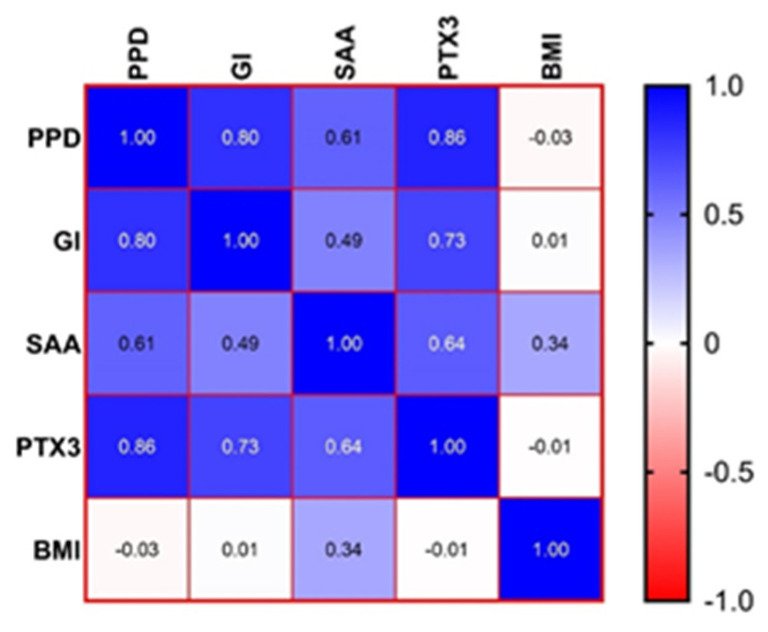
Heatmap of the correlation matrix between the measured characteristics. PPD, periodontal probing depth; GI, gingival index; SAA, serum amyloid A; PTX3, Pentraxin 3; BMI, body mass index.

The matrix correlation between the PPD, GI, SAA, PTX3, and BMI values is presented in Figure 1, underlying the strong positive correlations between the PPD and GI, PPD and PTX3, and the GI and PTX3.

## 4. Discussion

Using the Pentraxin 3 and serum amyloid A levels in GCF, this study aimed to assess the inflammatory state of periodontal tissues in obese patients. Periodontitis is caused by the interaction of subgingival microorganisms with the host’s complex immunity in response to infection. According to the findings of many studies, chronic periodontitis can be a factor in the development of a variety of systemic conditions [19]. Because obesity is a risk factor for increased morbidity and mortality in many diseases, including diabetes, cancers, and cardiovascular and other chronic diseases, the increased incidence of overweight and obese people around the world is a significant problem for the public’s health [20].

Previous clinical studies as well as systematic reviews have investigated the connections between obesity and PD. Because of the positive and consistent associations that have been found between the clinical parameters related to obesity and periodontitis, it has been hypothesized that people who are overweight or obese have an increased risk of periodontitis [21]. Several factors can explain why overweight or obese individuals are at a higher risk of developing periodontitis than those who are not. It was shown that adipose tissue is capable of, through its release of cytokines that are involved in inflammation processes, regulating several pathologic processes and functions [22].

Our research revealed statistically significant differences regarding the periodontal parameters between groups. The PPD values were significantly higher in the P + O group compared to group P, as well as the GI. In a study comparing the mean values of periodontal parameters among different categories of BMI, the authors found that a significant difference was obtained for all the periodontal parameters: the plaque index, the GI, and the PPD [23].

Akram’s study concluded that periodontal inflammation has a greater impact on the levels of biomarkers in GCF than obesity does, while whether patients having chronic periodontitis andobesity have elevated resistin, adiponectin, TNF-*α*, leptin, IL-6, IL-8, and IL-1*β* GCF levels compared to nonobese individuals remains debatable [24].

It has not yet been fully determined how SAA functions biologically, particularly about its rolein the metabolism of glucose and lipids. It has been suggested that the SAA protein, when associated with the HDL particles, may inhibit the transport of reverse cholesterol, and this protein has been related to the decreased levels of HDL that are seen during inflammation [25].

Our study showed that both groups of periodontitis patients (P and P + O) expressed significantly higher levels for SAA and PTX3. Temelli et al. showed significant correlations with PTX3 and SAA in a study based on the relationship between coronary artery disease, the values of SAA, PTX3, and the periodontal inflamed surface even though the patients had no coronary disease [26]. In a review by Zhao et al., it was shown that there have been studies conducted on the relationship between the SAA and other inflammatory markers or the relationship between SAA and obesity or related disorders, including cardiovascular disease, atherosclerosis, diabetes, and insulin resistance, highlighting a strong association between body mass index and SAA levels [27].

In our research, the SAA value was statistically significantly higher in all the test groups than in group C, with the highest values in the P + O group. Similarly, Türer et al. showed that the SAA concentrations in serum and gingival crevicular fluid in patients with chronic periodontitis were elevated compared to periodontally healthy individuals [28]. Although there was a weakly positive correlation between the SAA values and BMI in our study, to the limit of significance, Yang’s findings indicated that the serum SAA levels correlated with the BMI and fat mass in subjects who were overweight or obese before they lost weight. In addition, the correlation between the systemic SAA and total body fat mass was still present even after the participants had reduced their body fat percentage. These observations lend even more credence to the theory that BMI as well as fat mass is a factor in determining the circulating levels of SAA. No correlation was found between the changes in BMI and the changes in serum SAA in the analysis performed by Yang et al. [29]. According to a meta-analysis, the BMI levels and SAA levels were positively correlated, and the SAA levels decreased with weight loss [27]. A significantly positive correlation between the SAA and BMI levels was found in additional studies. Other authors have found by a quantitative meta-analysis that SAA, a biomarker of acute inflammation, has a substantial correlation with obesity [15,30,31].

In a 2015 study, Ardila et al. observed that patients with chronic periodontitis had higher serum levels of SAA than individuals without periodontitis [32]. A series of studies have published similar results, finding that the level of the SAA was elevated in patients with marginal or apical periodontal inflammation [32,33,34]. Similarly, patients with periodontitis had significantly higher levels of high-sensitivity C-reactive protein (hs-CRP) than healthy individuals. The SAA showed a positive correlation with PD and revealed a positive correlation with CAL [32].

A recent study on obese children found high PTX3 levels and a significant correlation with BMI [35]. Obesity causes low-grade inflammation mostly due to adipocytes and immune cells secreting proinflammatory molecules such astumor necrosis factor receptors 1 and 2, PTX3, and Il15. Obesity increased the PTX3 levels, according to the same study [36].

We found higher values of PTX3 in all the test groups than those found in the control group, with a statistically significant difference, with the highest values in the P + O group. In a 2019 study, the levels of PTX3 were found to be significantly higher in patients who had chronic periodontitis at the beginning of the study. According to Mohan [37], the PTX3 levels in gingival crevicular fluid increased with the severity of the inflammatory response, regardless of other systemic diseases. This could help identify patients predisposed to destructive diseases [38]. In our study, PTX3 was correlated strongly and significantly with the GI and very strongly and significantly with the PPD. In Fujita’s study, the mean clinical parameters of the GI, PPD, BOP, and GCF volume correlated positively with the mean PTX3 level [39]. Other studies with obese participants found that low PTX3 levels led to chronic inflammation in overweight and obese people. The PTX3 levels were higher in normal-weight people than in overweight and obese people, and the PTX3 levels were inversely related to the BMI [40]. We found no correlation between the PTX3 and BMI in our study. Karakas et al. [41] found that a lower body mass index and a smaller waist size have been associated with higher PTX3 concentrations. He also hypothesized that increasing PTX3 levels could be caused by metabolic syndrome components, which upregulate inflammation. In another study, increasing PTX3 levels were associated with metabolic syndrome indicators, including abdominal obesity [42]. These inhomogeneous results motivate further research to better understand the implication of these markers in the association of obesity and periodontitis.

The limitation of this study was the reduced number of participants derived from the exclusion criteria applied for greater accuracy of the study. Due to the small number of patients, we were unable to divide the periodontitis patients into groups based on the stage and degree of the periodontal disease.

## 5. Conclusions

Following the purpose of the study, patients with the obesity–periodontitis association had significantly higher levels of PTX3 and SAA than those with one of the diseases or healthy individuals, and these values correlated with certain clinical parameters.

## Figures and Tables

**Table 1:** Comparisons between the groups regarding PPD andGI.

Characteristics Mean (±SD) Median (IQR) Range	C	P	O	P + O	*p*-Value
PPD	0	3.43 (±0.94) 3.4 (2.75–3.95) 2.1–5.8	0	4.39 (±1.05) 3.9 (3.6–5.3) 2.9–6.4	P vs. P + O: 0.0156 *
GI	0	1.83 (±0.79) 1–3	0.94 (±0.68) 1 (0.25–1) 0–2	2.12 (±0.69) 2 (2–3) 1–3	P vs. O: 0.0025 ** P vs. P + O: 0.2957 O vs. P + O: <0.0001 ****

PPD, periodontal probing depth; GI, gingivalindex; C, control group; P, periodontitis group; O, obesity group; P + O, periodontitis and obesity group; * *p*-value < 0.05; ** *p*-value < 0.01; **** *p*-value < 0.0001.

**Table 2:** Statistical analysis for the SAA between groups.

Characteristics Mean (±SD) Median (IQR) Range	C	P	O	P + O	*p*-Value
SAA	1.87(±1.29) 2.75 (0.16–2.83) 0.17–3.0	4.98 (±0.99) 4.8 (4.11–5.7) 3.72–7	3.31 (±0.19) 3.25 (3.17–3.47) 3.06–3.69	15.74 (±13.16) 9.31 (7.78–16.54) 7.64–42.56	C vs. P: <0.0001 **** C vs. O: <0.0001 **** C vs. P + O: <0.0001 **** P vs. P + O <0.0001 **** P vs. O <0.0001 **** O vs. P + O <0.0001 ****

SAA, serum amyloid A; C, control group; P, periodontitis group; O, obesity group; P + O, periodontitis and obesity group; **** *p*-value < 0.0001.

**Table 3:** Statistical analysis for the PTX3 between groups.

Characteristics Mean (±SD) Median (IQR) Range	C	P	O	P + O	*p*-Value
PTX3	189.8 (±56.36) 220.3 (124.3–237.1) 101.4–239.5	383.4 (±76.89) 416.7 (280.5–446.7) 275.1–469.1	257.7 (±9.59) 256.2 (248.1–268.3) 244.3–275.1	505 (±24.43) 499 (484–524) 472.5–558.9	C vs. P: <0.0001 **** C vs. O: <0.0001 **** C vs. P + O: <0.0001 **** P vs. P + O <0.0001 **** P vs. O <0.0001 **** O vs. P + O <0.0001 ****

PTX3, Pentraxin 3; C, control group; P, periodontitis group; O, obesity group; P + O, periodontitis and obesity group; **** *p*-value < 0.0001.

## Data Availability

The data used to support the findings of this study are available from the corresponding author upon reasonable request.
